# Antibacterial Plant Secondary Metabolites: Mechanisms of Action, Pharmacokinetics, Safety, and Translational Potential

**DOI:** 10.3390/molecules31193477

**Published:** 2026-09-29

**Authors:** Ana Lizet Morales-Ubaldo, Adrian Zaragoza-Bastida, Lucía Delgadillo-Ruíz, Benjamín Valladares-Carranza, Abel Villa-Mancera, Eliazar Aquino-Torres, Rómulo Bañuelos-Valenzuela, Gabino Misael López-Rodríguez, Juan Noguez-Estrada, Jorge Vargas-Monter, Lenin Rangel-López, Nallely Rivero-Perez

**Affiliations:** 1 Área Académica de Medicina Veterinaria y Zootecnia, Instituto de Ciencias Agropecuarias, Universidad Autónoma del Estado de Hidalgo, Rancho Universitario Av. Universidad Km 1, EX-Hda de Aquetzalpa, Tulancingo 43600, Hidalgo, Mexico; mo260116@uaeh.edu.mx; 2 Unidad Académica de Medicina Veterinaria y Zootecnia, Universidad Autónoma de Zacatecas, Zacatecas 98068, Zacatecas, Mexico; luciadelgadillo@uaz.edu.mx (L.D.-R.); romulob@uaz.edu.mx (R.B.-V.); 3 Centro de Investigación y Estudios Avanzados en Salud Animal, Facultad de Medicina Veterinaria y Zootecnia, Universidad Autónoma del Estado de México, Km 15.5 Carretera Panamericana Toluca-Atlacomulco, Toluca 50200, Estado de México, Mexico; bvalladaresc@uaemex.mx; 4 Facultad de Medicina Veterinaria y Zootecnia, Benemérita Universidad Autónoma de Puebla, Tecamachalco 75482, Puebla, Mexico; abel.villa@correo.buap.mx; 5 Área Académica de Ciencias Agrícolas y Forestales, Instituto de Ciencias Agropecuarias, Universidad Autónoma del Estado de Hidalgo, Rancho Universitario Av. Universidad Km 1, EX-Hda de Aquetzalpa, Tulancingo 43600, Hidalgo, Mexico; eaquino@uaeh.edu.mx; 6 Universidad de la Costa, Carretera Libramiento Paraje Las Pulgas s/n, Colonia Cuauhtémoc, Santiago Pinotepa Nacional 71600, Oaxaca, Mexico; misael.rodriguez@uncos.edu.mx; 7 Ingeniería en Producción Animal, Universidad Politécnica de Francisco I. Madero, Domicilio Conocido Tepatepec, Francisco I. Madero 42660, Hidalgo, Mexico; jnoguez@upfim.edu.mx (J.N.-E.); jvargas@upfim.edu.mx (J.V.-M.); 8 Ingeniería en Acuacultura, División Académica de Ciencias Agropecuarias (DACA), Universidad Juárez Autónoma de Tabasco (UJAT), Villahermosa 86298, Tabasco, Mexico; lenin.rangel@ujat.mx

**Keywords:** plant secondary metabolites, antibacterial compounds, antimicrobial resistance, mechanisms of action, pharmacokinetics, biological safety, therapeutic translation

## Abstract

Antimicrobial resistance (AMR) has intensified the need for antibacterial agents with alternative chemical scaffolds and mechanisms of action. Plant-derived antibacterial compounds represent a chemically diverse source of bioactive molecules, but their therapeutic potential depends not only on in vitro potency but also on mechanistic validation, pharmacokinetic exposure, pharmacodynamic relationships, and biological safety. This review critically examines evidence published from 2014 to 2026 on major classes of plant-derived antibacterial compounds, emphasizing bacterial susceptibility, mechanisms of action, pharmacokinetics, and safety. Reported antibacterial effects include alterations in the cell envelope and membrane, disruption of energy metabolism, inhibition of protein synthesis and nucleic acid-associated targets, efflux and resistance modulation, and interference with quorum sensing, biofilm formation, and virulence. However, mechanistic evidence varies substantially among studies. Pharmacokinetic data reveal marked heterogeneity in absorption, bioavailability, tissue distribution, metabolism, protein binding, and elimination, while quantitative integration of antibacterial potency with active exposure and PK/PD remains limited. Cytotoxicity and in vivo toxicity studies further indicate that selectivity and tolerability are compound-, formulation-, dose-, and model-dependent. Overall, therapeutic translation requires integration of chemically defined antibacterial activity, validated mechanisms, infection-site exposure, PK/PD, host–cell selectivity, systemic safety, and efficacy in relevant infection models.

## 1. Introduction

Bacterial infections are becoming increasingly difficult and, in some cases, impossible to treat, because of antimicrobial resistance (AMR), which is recognized by international health organizations as one of the greatest global threats to public health [1]. The emergence and spread of AMR are influenced by multiple factors, including the misuse and overuse of antimicrobials in human and veterinary medicine, inadequate diagnostic testing, the extensive use of antimicrobials in agriculture and food production, and the limited development of novel antibacterial agents [2].

The search for alternative antibacterial therapies has therefore become a research priority. In particular, the development of antibacterial agents with novel or alternative mechanisms of action is of considerable interest because they may expand the current therapeutic options and potentially overcome existing resistance mechanisms [3,4]. In this context, plant secondary metabolites have attracted considerable attention because of their chemical diversity, multiple biological activities, and ability to interact with different bacterial targets [5].

Medicinal plants have been used for centuries in traditional medicine to prevent and treat infectious diseases. Knowledge regarding their therapeutic applications has been transmitted across generations in many cultures around the world [6]. According to the World Health Organization (WHO), more than 100,000 medicinal plant species are distributed across 91 countries, and herbal medicines are widely recognized for their accessibility, affordability, and therapeutic potential [7,8,9].

Plants synthesize a broad diversity of secondary metabolites, which encompass several chemical groups, including terpenes, phenolic compounds, alkaloids, and other structurally distinct constituents which are responsible for many of their biological properties, including antibacterial activity [3,10]. These compounds exert their antibacterial effects through multiple mechanisms, such as disruption of bacterial cell walls and membranes, inhibition of protein synthesis, interference with energy metabolism, inhibition of nucleic acid synthesis, and modulation of bacterial virulence and resistance factors [11,12,13,14].

However, the available evidence is highly heterogeneous. Some studies primarily report quantitative antibacterial activity, whereas others investigate mechanisms of action, pharmacokinetic behavior, toxicity, or in vivo efficacy; these evidence domains are not always available for the same compound. Consequently, antibacterial activity or a plausible mechanism of action should not be interpreted independently as evidence of therapeutic potential. Where sufficient data are available, antibacterial potency should be considered together with pharmacokinetic and pharmacodynamic information, including systemic and tissue exposure, to assess whether biologically relevant concentrations can realistically be achieved in vivo [15,16,17].

Previous reviews have extensively summarized plant-derived antibacterial compounds and their proposed mechanisms of action. Nevertheless, a critical comparison of the strength and completeness of the evidence across antibacterial activity, mechanistic characterization, pharmacological exposure, safety, and in vivo efficacy remains necessary. Such an approach can distinguish compounds supported mainly by in vitro findings from those for which complementary mechanistic and pharmacological evidence provides a stronger basis for further development.

Therefore, this critical narrative review evaluates the available evidence on plant-derived antibacterial compounds by considering antibacterial activity, mechanisms of action, pharmacokinetic and pharmacodynamic properties, safety, and in vivo efficacy when such information is available. Attention is given to differences in bacterial susceptibility, the strength of experimental support for proposed mechanisms, and the extent to which antibacterial potency can be related to pharmacological exposure. The main limitations and knowledge gaps that currently hinder the development of these compounds as antibacterial agents are also discussed.

## 2. Literature Search and Scope

This study is a narrative literature review designed to provide a broad and critical overview of current knowledge on the antibacterial activity, mechanisms of action, pharmacokinetics, pharmacodynamic considerations, safety, and translational potential of plant secondary metabolites. Therefore, systematic review methodology and reporting guidelines (e.g., PRISMA) were not applied.

Literature research was conducted in PubMed, Scopus, and ScienceDirect for studies published from 2014 to 2026. Boolean operators (AND/OR) were used to combine terms related to plant secondary metabolites and antibacterial activity. The main search strategy included (“plant secondary metabolites” OR phytochemicals OR “plant-derived antibacterial compounds” OR terpenes OR terpenoids OR phenolics OR flavonoids OR alkaloids OR “essential oils”) AND (antibacterial OR antimicrobial) AND (“mechanism of action” OR membrane OR efflux OR “quorum sensing” OR biofilm OR virulence). Additional searches combined plant-related terms with (pharmacokinetics OR ADME OR bioavailability OR metabolism OR “protein binding” OR pharmacodynamics OR “PK/PD” OR cytotoxicity OR toxicity OR safety). Search syntax was adapted to each database, and targeted searches were performed for individual compounds and specific mechanistic endpoints. Backward and forward citation searching was also used to identify additional relevant studies.

The literature search was last updated on July 2026. Search syntax was adapted to the requirements of each database while maintaining the same conceptual search structure and publication period. Because this work was designed as a critical narrative review rather than a systematic review, records were not processed through a formal deduplication and screening workflow; therefore, PRISMA-style counts of initially identified, excluded, and finally included records were not prospectively recorded.

Primary experimental studies were prioritized for quantitative antibacterial, mechanistic, pharmacokinetic, and safety data, whereas review articles were mainly used for contextual information and identification of the additional primary literature. Purified compounds, essential oils, extracts, fractions, and formulations were distinguished because their chemical complexity and experimental interpretation differ. Accordingly, antibacterial activity, mechanistic effects, pharmacokinetic parameters, and safety findings were interpreted as specific to the material evaluated, and results obtained with complex preparations were not attributed to individual constituents unless those constituents were independently assessed. Mechanistic evidence was critically interpreted according to the experimental approach used, distinguishing direct functional or biochemical evidence from supportive morphological observations, indirect molecular evidence, and computational predictions.

Botanical nomenclature and author citations were verified using World Flora Online (WFO) and, when necessary, the International Plant Names Index (IPNI). Accepted botanical names were used throughout the manuscript, and botanical authorities were provided at the first mention of each species.

ChemDraw Ultra 12.0.2 (CambridgeSoft, Cambridge, MA, USA) was used to draw the chemical structures.

## 3. Secondary Metabolites

Plants produce two major groups of metabolites: primary and secondary metabolites. Primary metabolites are essential for plant growth, development and reproduction, as they participate in fundamental physiological processes. In contrast, secondary metabolites (SMs) are involved in ecological interactions and plant adaptation, including defense against pathogens, herbivores, and environmental stress [18].

**Figure 1:**
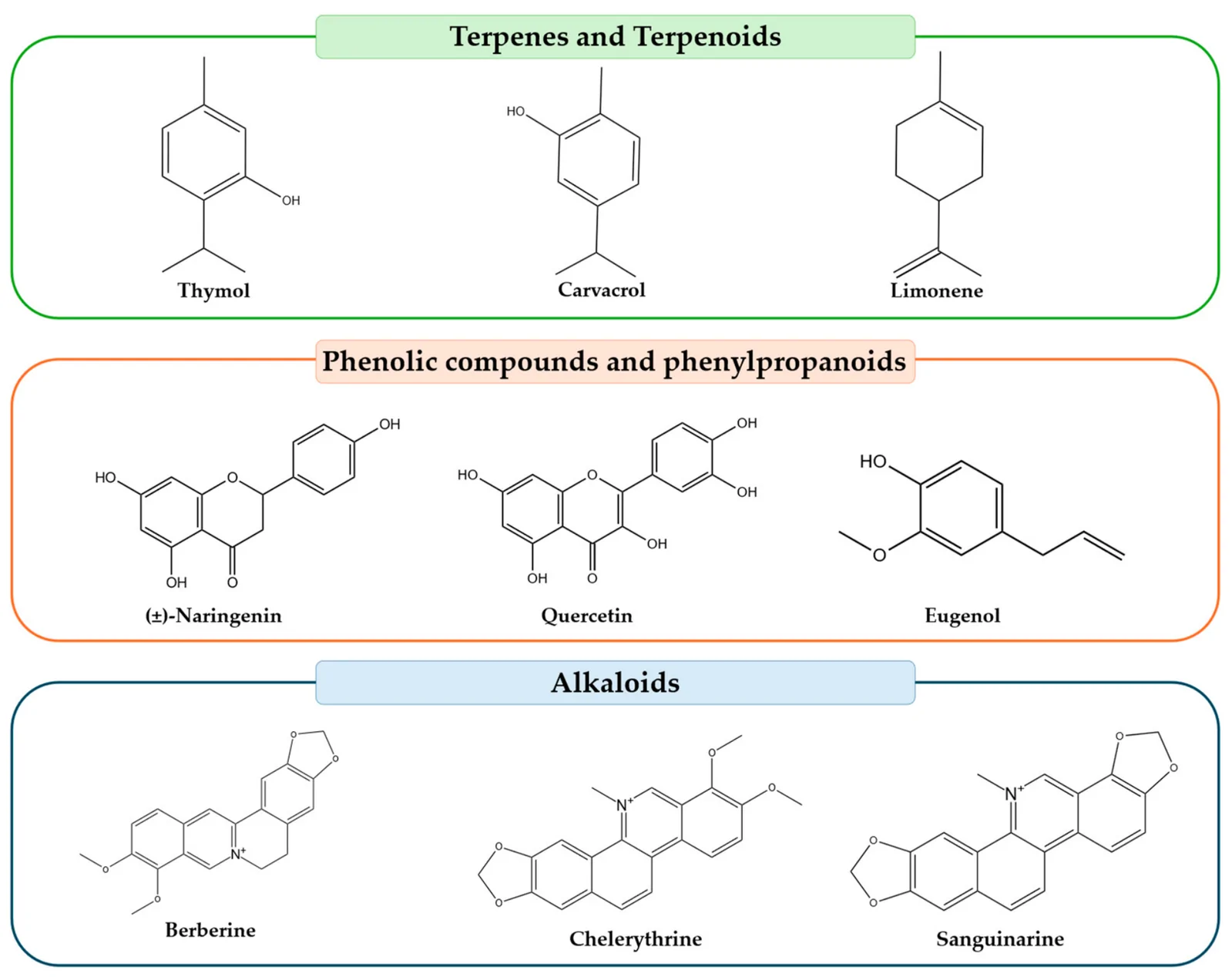
Representative chemical structures of antibacterial plant secondary metabolites from the main chemical classes discussed in this review. Names were verified, and stereochemistry is shown where applicable.

Plant secondary metabolites comprise chemically diverse groups that can be classified according to their biosynthetic origin, chemical structure, functional groups, and physicochemical properties [7,18,19]. Among the major classes associated with antibacterial activity are terpenes and terpenoids, phenolic compounds, phenylpropanoids, and alkaloids, although additional structurally distinct groups may also contribute to the antibacterial properties of medicinal plants (Figure 1).

The chemical diversity of these metabolites is closely associated with their broad spectrum of biological activities, including antibacterial activity. Reported antibacterial effects include disruption of bacterial cell walls and membranes, inhibition of essential enzymes and bacterial toxins, modulation of quorum sensing, biofilm formation, and virulence-associated processes [20,21]. However, these effects are not exclusive to a particular chemical class, and structurally distinct metabolites may converge on similar bacterial targets.

Accordingly, the following section examines the main classes of plant-derived antibacterial metabolites within a unified framework, emphasizing their structural characteristics, antibacterial activity, and the experimental evidence supporting their proposed mechanisms of action.

In this review, the term plant-derived antibacterial compounds is used to refer broadly to plant secondary metabolites for which antibacterial activity has been reported. When appropriate, isolated compounds, essential oils, extracts, and fractions are distinguished because their chemical complexity and experimental interpretation differ substantially.

## 4. Major Classes of Plant-Derived Antibacterial Compounds

Plant-derived antibacterial compounds encompass chemically diverse secondary metabolites with distinct structural and physicochemical properties that influence their biological activity. Among the classes most frequently investigated for antibacterial effects are terpenes and terpenoids, phenolic compounds, alkaloids, and phenylpropanoids [22,23]. These compounds may be evaluated as isolated molecules or as constituents of chemically complex materials, including essential oils and plant extracts [22]. Because their chemical complexity may influence antibacterial activity and mechanistic interpretation, isolated compounds, essential oils, and plant extracts should be distinguished when comparing experimental evidence [22].

### 4.1. Terpenes and Terpenoids

Terpenes and terpenoids are among the largest and most diverse groups of plant secondary metabolites and constitute the major bioactive components of many essential oils (EOs). Strictly, terpenes are hydrocarbons composed of isoprene-derived units, whereas terpenoids are structurally related derivatives containing oxygenated or other functional modifications. These compounds are volatile, hydrophobic molecules responsible for the characteristic aroma of numerous aromatic plants and play important ecological roles in plant defense against microorganisms, herbivores, and environmental stress. Due to their structural diversity and lipophilic nature, terpenes exhibit a broad spectrum of biological activities, particularly antibacterial activity [7,10,22,24].

The antibacterial activity of essential oils cannot be attributed to a single mechanism, because they consist of complex mixtures of bioactive constituents. Nevertheless, their activity is frequently associated with interaction of hydrophobic components with bacterial membranes, although additional intracellular and regulatory effects have also been reported [25,26,27].

### 4.2. Phenolic Compounds

Phenolic compounds constitute one of the largest and most diverse groups of plant secondary metabolites and are widely distributed in leaves, fruits, seeds, and other plant tissues. Based on their chemical structure, they include several subclasses, among which flavonoids, phenolic acids and tannins are frequently investigated for antibacterial activity [28,29].

Flavonoids represent a major subgroup and include flavones, flavonols, flavanones, flavanols, isoflavones, dihydroflavonols, chalcones, and biflavonoids. Representative antibacterial flavonoids include kaempferol, naringenin, quercetin, glabrol, KCR, and baicalin, which have been investigated against different bacterial species through diverse experimental approaches [13,30,31,32,33,34,35].

Phenolic acids and related compounds, including vanillic acid, protocatechuic acid, and catechin, have also demonstrated antibacterial and antibiofilm effects [36]. Tannins comprise another relevant group and include condensed tannins, persimmon tannins, and hydrolysable tannins such as PGG and dGVG, which have been evaluated for antibacterial and antivirulence activities [37,38,39,40]. Tannic acid has been investigated for its effects on quorum sensing and virulence-associated processes [41]. Overall, phenolic compounds exhibit broad structural and functional diversity. Their antibacterial effects involve both cell-envelope and intracellular processes; however, these mechanisms are discussed comparatively in Section 5 to distinguish the type and strength of experimental evidence supporting each proposed target.

### 4.3. Alkaloids

Alkaloids comprise a structurally diverse group of nitrogen-containing secondary metabolites with a broad range of biological activities. Based on their biosynthetic origin and structural characteristics, they are commonly classified as true alkaloids, protoalkaloids, and pseudoalkaloids, with numerous additional structural subclasses. Several plant-derived alkaloids have demonstrated antibacterial activity against susceptible and multidrug-resistant bacteria [42,43].

Protoberberine alkaloids represent an important group of antibacterial alkaloids. Compounds such as pseudocolumbamine, jatrorrhizine, palmatine, 4,13-dihydroxy-3,9,10-trimethoxyprotoberberine, and 13-hydroxy-2,3,9,10-tetramethoxyprotoberberine, isolated from *Annickia chlorantha* (Oliv.) Setten & Maas have been investigated for their effects on bacterial growth and energy metabolism [44]. Berberine is among the most extensively studied plant-derived alkaloids and has been evaluated for antibacterial, antibiofilm, quorum-sensing-modulating, and efflux-pump-inhibitory activities [45,46,47].

Other representative alkaloids include chelerythrine, which has demonstrated antibacterial activity associated with alterations in bacterial membrane integrity and protein biosynthesis [48,49], and palmatine, which has been investigated for interactions with bacterial DNA [50]. Collectively, these findings illustrate the diversity of bacterial processes affected by alkaloids; however, the experimental evidence supporting individual mechanisms varies considerably and is discussed comparatively in Section 5.

Taken together, these three major chemical groups differ substantially in their structural and physicochemical characteristics, but their antibacterial effects frequently converge on common bacterial processes. The following section therefore compares these mechanisms across chemical classes rather than treating each class independently.

## 5. Antibacterial Mechanisms and Bacterial Determinants of Susceptibility

Plant-derived antibacterial compounds affect multiple bacterial structures and physiological processes, including the cell envelope, energy metabolism, efflux systems, quorum sensing and biofilm formation, among others [11,22,51]. However, susceptibility to these compounds depends not only on their chemical characteristics and concentration but also on intrinsic bacterial features, particularly cell-envelope architecture. Therefore, mechanistic evidence should be interpreted in relation to the bacterial species and experimental model in which each effect was demonstrated [52].

### 5.1. Gram-Dependent and Compound-Dependent Variation in Antibacterial Susceptibility

Susceptibility to plant-derived antibacterial compounds is influenced by compound properties and intrinsic bacterial characteristics. A major determinant is cell-envelope architecture. Gram-negative bacteria possess a lipopolysaccharide outer membrane that constitutes an additional barrier and can restrict the access of antibacterial molecules to cytoplasmic membrane and intracellular targets [51]. Permeation across the Gram-negative outer membrane is influenced by the physicochemical properties and may occur through different routes, including diffusion through porins for small hydrophilic molecules and lipid-mediated pathways for more hydrophobic compounds. Differences in outer-membrane composition, porin characteristics, and active efflux can therefore affect compound accumulation and bacterial susceptibility [53].

This structural distinction has frequently been used to explain the greater susceptibility of Gram-positive bacteria to plant-derived antibacterial compounds. However, this tendency should not be considered universal. Different studies have identified greater activity against Gram-positive bacteria and studies in which no clear Gram-dependent difference was observed [52,53].

Andrade-Ochoa et al. (2021) [52] compared seven essential oils and eight pure compounds against three Gram-positive and three Gram-negative bacterial species. Although a tendency toward lower MIC values against Gram-positive bacteria was observed for several essential oils, the differences observed varied according to the tested oil. For example, cumin oil exhibited lower MIC values against *B. cereus* and *S. aureus* (150–200 µg/mL) than against *E. coli*, *S. typhimurium* and *S. sonnei* (600 µg/mL). In contrast, oregano oil exhibited comparable or lower MIC values against the Gram-negative species (66–100 µg/mL) than against the Gram-positive species (83–100 µg/mL).

A similar compound-dependent pattern was observed for isolated essential oil constituents. Thymol exhibited consistently low MIC values against both Gram-positive and Gram-negative bacteria, ranging from 23 to 91 µg/mL and from 25 to 41 µg/mL, respectively. Carvacrol also showed substantial activity against both bacterial groups, although susceptibility varied among species. In contrast, eucalyptol showed markedly higher MIC values against the Gram-negative bacteria evaluated (466–666 µg/mL) than against most Gram-positive species (83–416 µg/mL). Other compounds showed no consistent Gram-associated pattern; for example, cuminaldehyde exhibited a lower MIC against *E. coli* (50 µg/mL) than against *B. cereus* or *L. monocytogenes*, whereas limonene displayed wide interspecies variability within both Gram groups. Thus, even among structurally related or hydrophobic constituents, bacterial susceptibility appears to reflect interactions between molecular properties and species-specific bacterial characteristics rather than a uniform Gram-dependent response [52].

A similar heterogeneous pattern has been reported for polyphenolic compounds against *E. coli* O157, *S. aureus*, *S. Typhimurium*, *Enterobacter sakazakii*, and *Vibrio parahaemolyticus*. The antibacterial activity varied according to both compound and bacterial species. Baicalein showed relatively low MIC values across the bacterial panel (0.5–1.5 mmol/L), whereas its derivative, baicalin, exhibited substantially weaker activity, with MIC values of 2.5 or >2.5 mmol/L. Myricetin also showed species-dependent activity, with MIC values of 0.5 mmol/L against *E. coli* and *Vibrio parahaemolyticus* but 1.0–2.5 mmol/L against remaining bacteria, including *S. aureus*. In contrast, quercetin displayed relatively similar MIC values across most evaluated bacteria (1.0–2.0 mmol/L). Comparisons between structurally related polyphenols further indicated that glycosylation could modify antibacterial activity. Glycosylated flavonoids generally showed lower activity than their corresponding aglycones in this experimental series; notably, antibacterial activity of resveratrol was lost after substitution of the hydroxyl group at C-3 with a glucosyl group to form piceid [54]. These findings illustrate that bacterial susceptibility depends not only on Gram status but also on specific structural modifications of the antibacterial scaffold.

Structure–activity studies of flavonoids further support this interpretation. Echeverria et al. (2017) [55] evaluated eight naturally occurring flavones and flavanones against four Gram-positive and four Gram-negative bacterial species and demonstrated that antibacterial activity was related to both bacterial group and compound physicochemical properties. Galangin was the most active flavonoid evaluated, whereas progressive O-methylation of hydroxyl groups reduced antibacterial activity: 3-*O*-methylgalangin showed lower activity than galangin, and 3,7-*O*-dimethylgalangin became inactive. Active flavonoids against Gram-negative bacteria also occupied a narrower lipophilicity range than compounds active against Gram-positive bacteria. Importantly, these findings do not indicate that increasing lipophilicity intrinsically enhances antibacterial activity. Rather, the authors identified an amphipathic balance in which both hydrophilic and hydrophobic structural features contribute to activity. Thus, antibacterial potency appears to depend on the combined effects of hydroxylation, methylation, and lipophilicity rather than on any one physicochemical characteristic alone.

The importance of the position of hydroxyl groups, rather than simply their number, is further supported by Hummelova et al., who evaluated 15 naturally occurring isoflavones and their metabolites against Gram-positive and Gram-negative bacteria, and found marked differences in antibacterial activity among structurally related compounds. Their structure–activity relationship (SAR) analysis identified ortho-dihydroxyisoflavones as particularly active and highlighted hydroxyl substitution at the C-5, C-6, and C-7 positions as relevant to antibacterial activity. Demethyltexasin showed the strongest activity, particularly against *S. aureus*, including resistant isolates, while some metabolites exhibited greater antibacterial activity than their parent compounds. These findings demonstrate that hydroxylation pattern and substitution position can substantially influence antibacterial potency within the same chemical class [56].

A similar pattern has been reported among alkaloids. In a comparative study of benzophenanthridine alkaloids from *Macleaya cordata* (Willd.) R.Br., sanguinarine, 6-ethoxysanguinarine, and 6-methoxydihydrosanguinarine exhibited activity against both Gram-positive and Gram-negative bacteria, whereas chelerythrine and dihydrochelerythrine showed weaker activity against Gram-negative species; however, dihydrochelerythrine retained antibacterial activity against *S. aureus*. Further evaluation against clinical MRSA isolates revealed marked potency differences among these structurally related alkaloids, with 6-ethoxysanguinarine showing the greatest overall activity. Comparative SAR analysis associated the 6-ethoxy and 7,8-methylenedioxy substitution patterns with enhanced anti-MRSA activity [57]. These findings extend the SAR beyond flavonoids and indicate that specific substituent patterns can modify antibacterial potency and spectrum within structurally related alkaloids.

Nevertheless, greater susceptibility of Gram-positive bacteria is not consistently observed among alkaloids. Tetrandrine and limacusine, isolated from *Phaeanthus ophthalmicus* (Roxb. ex G.Don) J. Sinclair, exhibited MIC and MBC values of 68.75 µg/mL against carbapenem-resistant *Klebsiella pneumoniae* and metallo-β-lactamase-producing *Pseudomonas aeruginosa*, whereas values of 137.5 µg/mL were reported against MRSA and vancomycin-resistant *Enterococcus* spp. Thus, under the conditions evaluated, the Gram-negative isolates were more susceptible to these alkaloids than Gram-positive isolates. This contrasting response further indicates that the Gram-negative outer membrane does not invariably predict reduced susceptibility to plant-derived antibacterial compounds [58].

Collectively, these comparative studies demonstrate that antibacterial SAR cannot be reduced to a single structural determinant. Hydroxylation pattern, O-methylation, glycosylation, specific alkoxy or methylenedioxy substitutions, and lipophilicity can modify antibacterial potency, but their effects depend on the underlying molecular scaffold and the bacterial species evaluated. Thus, Gram status contributes to susceptibility but does not independently predict antibacterial potency; the observed response reflects the interaction between bacterial-envelope characteristics, species- and strain-specific factors, and the structural and physicochemical properties of individual plant-derived compounds [54,55,56,57,58].

### 5.2. Cell Envelope and Membrane Disruption

Disruption of the bacterial cell envelope is among the most frequently reported mechanisms for plant-derived compounds [22,59]. Reported membrane-associated effects include changes in permeability and membrane potential, alterations in membrane fluidity, and leakage of intracellular constituents [59,60]. Nevertheless, the experimental support of these effects is heterogeneous, and the proposed mechanism should be interpreted according to the type of evidence generated [59].

Evidence from essential oils and essential oils-based formulations illustrates this mechanistic diversity; *Persicaria orientalis* (L.) Spach essential oil induced morphological alterations and loss of cellular integrity in *Pectobacterium carotovorum* [61]. More direct functional evidence was reported for cinnamon essential oil nanoemulsion against *Pseudomonas deceptionensis,* where propidium iodide and NPN uptake, membrane potential measurements, and leakage of proteins and electrolytes demonstrated disruption of membrane integrity and permeability [62]. *Litsea cubeba* (Lour.) Pers. essential oil also produced morphological damage in *Cutibacterium acnes* [63]. Meanwhile, *Citrus limon* (L.) *Osbeck.* essential oil induced membrane damage accompanied by leakage of intracellular proteins and nucleic acids [64]. Similarly, garlic (*Allium sativum* L.) essential oil nanoemulsion caused membrane depolarization, loss of membrane integrity, and leakage of nucleic acids, proteins, and ATP in *Listeria monocytogenes* [65]. Comparable membrane-associated effects have been reported for chemically characterized clove essential oil against *S. aureus* and *P. aeruginosa* [66]. Importantly, because these materials are chemically complex essential oils or formulations, their effects cannot be unequivocally attributed to an individual constituent.

Studies with isolated essential oil constituents provide more compound-specific evidence. Thymol and carvacrol altered membrane function in both *S. aureus* and *Acinetobacter baumannii*, although the magnitude of depolarization and permeabilization differed according to the compound and bacterial species [67]. Eugenol also produced loss of membrane integrity in carbapenem-resistant *K. pneumoniae*, accompanied by leakage of DNA, proteins, and β-galactosidase; however, these effects occurred together with increased reactive oxygen species, suggesting that membrane damage formed part of a broader oxidative antibacterial response [68]. Thus, even for isolated constituents, membrane disruption should not necessarily be interpreted as the only antibacterial mechanism.

Membrane-associated effects have also been reported for phenolic compounds, although the strength of the supporting evidence varies among studies. Naringenin and quercetin produced marked morphological alterations in mycobacterial cells, including surface depressions, irregular morphology, and loss of the peptidoglycan layer [33]. These observations are predominantly morphological; they support cell-envelope damage but do not by themselves establish a direct membrane target. In contrast, glabrol increased membrane permeability and disrupted the proton motive force in *S. aureus*, providing functional evidence of membrane-associated antibacterial activity [34]. Catechin has also been associated with increased membrane permeability [36]. Meanwhile, persimmon tannins produced broader alterations involving membrane transport, permeability, and intracellular pH homeostasis [38].

More recent studies provide additional functional evidence for flavonoids. Faleye et al. reported that selected flavonoid derivatives, particularly 3,2-dihydroxyflavone, altered membrane permeability in *Vibrio parahaemolyticus* while simultaneously affecting metabolic activity, cell division, biofilm formation, and virulence-associated processes [69]. Likewise, quercetin induced concentration-dependent membrane damage in *S. aureus* ATCC 27217, accompanied by leakage of proteins and nucleic acids. Integrated metabolomic and transcriptomic analysis further showed alterations in membrane fatty-acid composition, while enzyme assays demonstrated inhibition of FabG, indicating that membrane dysfunction was associated with interference in fatty-acid biosynthesis rather than simple physical membrane rupture [70]. These findings indicate that membrane dysfunction may represent one component of a broader, multitarget antibacterial response rather than an isolated mechanism.

Hydrolysable tannins further illustrate that membrane-associated activity does not necessarily involve extensive membrane rupture. Penta-*O*-galloyl-β-D-glucose (PGG) and 1,2-di-*O*-galloyl-4,6-valoneoyl-β-D-glucose (dGVG) altered membrane fluidity in *S. aureus* without causing complete membrane disruption [39]. These compounds also affected membrane-associated processes, including membrane-vesicle production and interactions with α-hemolysin [39,40]. Thus, membrane effects produced by phenolic compounds may range from structural alterations and changes in fluidity to increased permeability and leakage, and these outcomes should not be considered mechanistically equivalent.

Regarding alkaloids, berberine has been reported to alter the cell envelope of MRSA, producing concentration-dependent changes in cell-wall structure and membrane integrity [46]. More direct functional evidence was obtained by Zhao et al., who showed that berberine increased membrane permeability and disrupted the proton motive force in methicillin-susceptible and methicillin-resistant *S. aureus*, as assessed using propidium iodide and DiSC3(5) [71]. However, this bacterial effect was strongly dependent on alkaline conditions (pH 8.5–9.0), indicating that the membrane-associated mechanisms should not be extrapolated uncritically to other physiological environments.

Chelerythrine provides additional functional evidence of cell-envelope damage. In carbapenem-resistant *Serratia marcescens*, chelerythrine altered membrane potential, intracellular pH, ATP levels, and membrane integrity [48]. Experiments in *S. aureus* and MRSA also demonstrated increased alkaline-phosphatase and intracellular-protein leakage, together with morphological alterations in the cell wall and membrane [49]. Nevertheless, inhibition of protein biosynthesis was simultaneously observed, indicating that membrane damage represents one component of a broader antibacterial response rather than an exclusive mechanism [49].

### 5.3. Energy Metabolism

Interference with bacterial energy metabolism has been reported for several plant-derived antibacterial compounds, although the supporting evidence ranges from direct biochemical measurements to indirect transcriptomic or metabolomic associations. The proton motive force, generated by the membrane potential and transmembrane proton gradient, contributes to ATP synthesis, active transport, and other energy-dependent cellular processes; consequently, disruption of the proton motive force of ATP generating pathways can impair bacterial viability [72].

Among essential oils, *L. cubeba* provides relatively strong experimental evidence of metabolic disruption. In *Cutibacterium acnes*, treatment with the essential oil significantly reduced intracellular ATP synthesis and altered the activity of key enzymes, including pyruvate carboxylase, malate dehydrogenase, pyruvate kinase, and hexokinase [63].

Metabolomic analysis further identified perturbations in carbohydrate and energy metabolism. These effects were accompanied by structural damage to bacterial cells; this mechanism cannot be considered independently from broader cell-envelope damage.

Other essential oils have been linked to energy metabolism mainly through omics-based evidence. Transcriptomic analysis of *Phoebe bournei* (Hemsl.) Yen C. Yang essential oil-treated *S. aureus* and *B. cereus* revealed differential expression of genes involved in energy metabolism, together with alterations in nucleotide, lipid, and membrane-associated pathways. Species-specific responses were also observed, including downregulation of genes related to K+ uptake in *S. aureus* and glycogen metabolism in *B. cereus*. These findings support metabolic involvement but should be interpreted as indirect mechanistic evidence because transcriptional changes do not by themselves establish functional inhibition of energy production [73].

Comparable effects have been reported for phenolic compounds. Kaempferol affected energy-metabolism-related pathways in *Xanthomonas oryzae*, as supported by transcriptomic, qRT-PCR, ultrastructural, and biochemical analyses [13]. However, its antibacterial response also involved disruption of cellular integrity and reduced pathogenicity, indicating a broader multitarget effect rather than selective inhibition of energy metabolism. Persimmon tannin similarly altered glycolysis, the tricarboxylic acid cycle, carbohydrate metabolism, and intracellular pH regulation in MRSA [38]. These results were derived primarily from transcriptomic and metabolomic analyses; therefore, they do not provide direct evidence of a specific enzymatic target.

More direct evidence of interference with bacterial bioenergetics has been obtained for some alkaloids. Protoberberine alkaloids isolated from *A. chlorantha* inhibited H^+^-ATPase-mediated proton pumping, as assessed through bacterial medium acidification assays [44]. This functional assay provides stronger support for disruption of energy-dependent processes. Berberine has also been shown to increase membrane permeability and collapse the PMF in methicillin-susceptible and methicillin-resistant *S. aureus* [71]. However, this effect was strongly dependent on alkaline conditions, indicating that its contribution to antibacterial activity may vary substantially with the physicochemical environment.

Current evidence indicates that plant-derived antibacterial compounds can interfere with bacterial bioenergetics at different levels, including ATP production, proton translocation, proton motive force maintenance, and central metabolic pathways. Nevertheless, decreases in ATP levels or changes in energy-metabolism-related gene expression should not automatically be interpreted as evidence of a direct metabolic target, because they may occur secondarily to membrane damage or broader cellular stress. Accordingly, direct measurements of ATPase activity, membrane potential, or ATP production provide stronger mechanistic support than transcriptomic or metabolomic alterations alone.

### 5.4. Protein and Nucleic Acid Targets

Interference with bacterial protein synthesis and nucleic-acid-associated processes represent another mechanism reported for plant-derived antibacterial compounds. However, the strength of mechanistic evidence varies considerably, ranging from functional translation and enzyme inhibition assays to proteomic responses and computational predictions. Accordingly, inhibition of protein or nucleic-acid metabolism should be distinguished from nonspecific reductions in macromolecular content that may occur secondary to membrane damage or generalized cellular stress [35,49].

Among phenolic compounds, 3-*O-α*-L-(2″,3″-di-*p*-coumaroyl) rhamnoside (KCR) provides direct experimental evidence of interference with bacterial translation. Proteomic analysis of KCR-treated *S. aureus* revealed extensive alterations in translation-associated proteins, including ribosomal proteins and proteins involved in rRNA and tRNA binding, tRNA processing, and aminoacyl-tRNA ligase activity [35]. Importantly, these proteomic observations were functionally validated using an in vitro transcription/translation assay, in which KCR inhibited luciferase production with an IC50 of 39.6 µg/mL [35]. Thus, unlike studies in which changes in ribosomal-gene expression are inferred to indicate translation inhibition, KCR provides functional evidence supporting protein synthesis as an antibacterial target.

Protein synthesis may also contribute to the antibacterial activity of some alkaloids. Chelerythrine reduced bacterial protein expression in *S. aureus* and MRSA, as demonstrated by changes in electrophoretic protein profiles [49]. However, the same study showed extensive cell-wall and membrane damage and extracellular protein leakage, indicating that inhibition of protein biosynthesis occurred together with major cell-envelope disruption rather than as an isolated mechanism [49]. Consequently, protein synthesis should be considered one component of the antibacterial response rather than an exclusive target of chelerythrine.

More direct evidence for interference with nucleic-acid-associated targets has been reported for flavonoids. Glabridin, an isoflavonoid from *G. glabra*, inhibited purified bacterial DNA gyrase with an IC50 of 0.8516 µmol/L and produced weaker inhibition of dihydrofolate reductase, with 29% inhibition at 100 µmol/L [74]. These effects were supported by DNA-damage assays, providing functional evidence that interference with DNA gyrase contributes to its antibacterial activity. Nevertheless, the substantially weaker effect on dihydrofolate reductase indicates that these two proposed targets should not be considered equivalent contributors to the antibacterial response [74].

Gallate derivatives provide additional evidence that structural variation can influence inhibition of bacterial topoisomerases. Digallic acid inhibited *E. coli* DNA gyrase with an IC50 of approximately 2 µmol/L and topoisomerase IV with an IC50 of approximately 8 µmol/L, whereas gallic acid itself showed little inhibition at concentrations up to 500 µmol/L. ATPase experiments indicated that digallic acid and related gallate derivatives act as ATP competitive inhibitors of DNA gyrase [75].

In contrast, evidence for some essential oils remains primarily predictive. Molecular docking of major constituents of *Cupressus funebris* (Endl.) essential oil, including citronellal and terpinen-4-ol, predicted interactions with bacterial DNA gyrase [76]. Because enzymatic inhibition or direct target engagement was not functionally demonstrated, these findings should be interpreted as evidence of a potential molecular target rather than proof of DNA-gyrase inhibition. This distinction is important when complex essential oils are evaluated, since antibacterial activity cannot be unequivocally assigned to a single constituent or intracellular target based on docking alone.

Overall, plant-derived compounds can interfere with bacterial translation and nucleic-acid-associated targets, but the strength of mechanistic evidence depends on the experimental approach. Functional translation or enzyme-inhibition assays provide stronger support than expression changes, DNA leakage, or docking alone.

### 5.5. Efflux Pumps and Resistance-Associated Mechanisms

Efflux pumps contribute to intrinsic and acquired antibacterial resistance by reducing the intracellular concentration of structurally diverse antibiotics. Several plant-derived compounds have been investigated as potential efflux-pump inhibitors or resistance-modifying agents; however, the strength of this evidence depends on the experimental approach. Increased accumulation of fluorescent pump substrates, restoration of antibiotic susceptibility in efflux-overexpressing strains, and changes in efflux-associated gene expression provide complementary evidence, whereas antibiotic potentiation alone does not establish direct inhibition of an efflux system [30,32].

Among phenolic compounds, fisetinidol showed no relevant intrinsic antibacterial activity but reduced the MIC of norfloxacin and ethidium bromide in *S. aureus* SA-1199B, a strain that overexpresses the NorA efflux pump [30]. These findings support a resistance-modifying effect consistent with NorA inhibition, although they do not demonstrate direct molecular binding to the pump. More direct phenotypic evidence was obtained for 2-phenylacetophenone and trans-chalcone, which increased intracellular ethidium bromide accumulation at subinhibitory concentrations in Gram-positive, Gram-negative and mycobacterial models [31]. Their efflux-associated effects were accompanied by additive or synergistic interactions with selected antibiotics, supporting their potential use as antibiotic adjuvants [31].

Flavonoid-mediated efflux modulation has also been demonstrated in mycobacteria. Skullcapflavone II and nobiletin increased ethidium-bromide accumulation in *Mycobacterium smegmatis* and *M. aurum*, with nobiletin showing strong efflux inhibitory activity [43]. Skullcapflavone II also markedly increased the susceptibility of *M. smegmatis* to ethidium bromide and rifampicin. However, the authors found no consistent relationship between the magnitude of efflux inhibition and antibiotic-modulating activity, indicating that increased antibiotic susceptibility cannot necessarily be attributed exclusively to efflux-pump inhibition [32].

Recent evidence further indicates that some terpenoid constituents can affect both efflux activity and resistance development. In *Acinetobacter baumannii*, thymol increased intracellular ethidium-bromide accumulation after acute exposure, whereas carvacrol required longer pre-exposure to produce a significant effect [67]. In 30-day serial-passage experiments, thymol and carvacrol alone did not produce substantial increases in MIC, and their combination with aminoglycosides attenuated the magnitude of resistance development compared with the antibiotics alone [67]. These findings provide direct experimental evidence that these compounds can modulate resistance evolution in vitro, although the contribution of efflux inhibition cannot be separated completely from their membrane, metabolic, and oxidative-stress effects.

Among alkaloids, berberine has also been associated with efflux-mediated resistance modulation. In a carbapenem-resistant clinical isolate of *Pseudomonas aeruginosa*, the combination of berberine and imipenem produced synergistic antibacterial activity (FICI = 0.375) and reduced expression of mexX, mexY, and oprM, components of the MexXY-OprM multidrug-efflux system [47]. These changes were observed during combined treatment, so they should not be interpreted as evidence that berberine alone directly binds to or inhibits the pump. Similarly, protoberberine alkaloids from *A. chlorantha* potentiated several antibiotics against MDR bacteria [44], but enhancement in their own activity by the reference efflux-pump inhibitor PAβN indicates involvement of bacterial efflux without demonstrating that these alkaloids themselves are direct efflux-pump inhibitors.

Interactions between plant-derived compounds and conventional antibiotics should be interpreted according to the experimental approach used. Checkerboard assays and FIC/FICI values provide quantitative assessment of concentration-dependent interactions, whereas time-kill experiments additionally characterize their temporal behavior. Therefore, reductions in antibiotic MIC alone should not be interpreted as definitive evidence of synergism, and additive or antagonistic interactions should also be considered when evaluating potential antibiotic adjuvants.

### 5.6. Quorum Sensing, Biofilm Formation, and Virulence

Plant-derived compounds can also interfere with bacterial communication, biofilm development, and virulence-associated factors. These effects should be interpreted separately because inhibition of biofilm formation does not necessarily demonstrate quorum-sensing (QS) inhibition, and changes in virulence-gene expression do not by themselves establish direct interaction with a QS regulator. Stronger evidence is obtained when phenotypic assays are combined with measurements of QS-related genes, signaling molecules, or virulence factors [77].

Among essential oils, clove oil inhibited biofilm formation and protease production in multidrug-resistant *P. aeruginosa* at subinhibitory concentrations [77]. RT-PCR analysis further showed reduced expression of *pelF*, involved in biofilm matrix formation, and *aprA*, which encodes an extracellular protease [77]. This study evaluated a chemically complex essential oil, so these effects cannot be attributed specifically to eugenol or to a single molecular target.

Phenolic compounds provide broader evidence of antivirulence activity. Baicalin reduced biofilm formation and virulence-associated gene expression in MRSA and improved survival and reduced bacterial burden in an infected-mouse model [14]. Thus, this study links in vitro modulation of virulence-related phenotypes with an in vivo outcome, although the contribution of individual mechanisms to therapeutic efficacy remains unresolved. In uropathogenic *E. coli*, a combination of catechin, vanillic acid, and protocatechuic acid also reduced adhesion to silicone surfaces through complementary effects on bacterial motility, surface properties, and membrane permeability [36].

Tannins also affect biofilm formation, motility, and virulence-associated processes. Condensed tannin extracts inhibited swimming, swarming, and biofilm formation in avian pathogenic *E. coli*, although the magnitude and direction of the response depended on tannin composition and concentration [37]. Low concentrations of some procyanidin-rich extracts increased attachment and motility, indicating that antibiofilm activity cannot be generalized across all tannin preparations [37].

Hydrolysable tannins act through additional antivirulence mechanisms. PGG and dGVG reduced membrane-vesicle production in *S. aureus* and interacted with α-hemolysin, whereas PGG also inhibited toxin secretion [39,40]. Tannic acid exhibited anti-QS and antivirulence activity against *S. enterica* Typhi and Paratyphi A, including reductions in extracellular polymeric substances, cell-surface hydrophobicity, and other QS-associated phenotypes [41]. These findings indicate that tannins may attenuate pathogenicity without necessarily requiring extensive bactericidal activity.

Among alkaloids, berberine hydrochloride provides relatively strong mechanistic evidence linking QS interference with biofilm inhibition. At sub-MIC concentrations, berberine hydrochloride reduced biofilm formation and swimming motility in *Hafnia alvei*; downregulated the QS-related genes *halI*, encoding an AHL synthase, and *halR*, encoding a LuxR-type QS transcriptional regulator; and decreased production of the signaling molecule C14-HSL [45]. Molecular docking and molecular-dynamic simulations further predicted interaction with the HalR receptor, although this computational evidence should be considered supportive rather than definitive proof of direct binding [56].

Chelerythrine also inhibited biofilm formation in carbapenem-resistant *Serratia marcescens*, including effects on biofilm components and preformed biofilms [48]. However, because chelerythrine simultaneously disrupts membrane function and cellular energetics, its antibiofilm activity should be interpreted as part of a broader antibacterial response rather than as evidence of a specific QS-targeting mechanism [48].

Overall, plant-derived compounds can attenuate biofilm formation and virulence through both QS-dependent and QS-independent mechanisms. Mechanistic confidence is greatest when phenotypic changes are supported by measurements of signaling molecules, QS-related genes, or defined virulence factors.

### 5.7. Strength and Limitations of Mechanistic Evidence

The mechanistic evidence supporting plant-derived antibacterial compounds varies substantially in strength. Functional assays that directly measure membrane potential or permeability, intracellular leakage, enzyme activity, protein synthesis, efflux, or quorum-sensing signals provide comparatively strong evidence for specific cellular effects. For example, functional validation has supported membrane-associated activity [34,67], inhibition of bacterial translation [35], and modulation of multidrug efflux [31,32,47]. Nevertheless, even these effects should not automatically be interpreted as exclusive primary targets, because several compounds simultaneously perturb multiple bacterial processes.

In contrast, morphological, transcriptomic, metabolomic, and computational approaches are better considered supportive or hypothesis-generating unless accompanied by functional validation. Morphological alterations can demonstrate cellular damage but do not identify its molecular cause, as illustrated by the envelope alterations reported for naringenin and quercetin [33]. Likewise, transcriptional or metabolic changes may reflect secondary responses to antibacterial stress rather than direct target inhibition [38,73]. Molecular docking provides an even more preliminary level of evidence: predicted interactions, such as those between *C. funebris* essential-oil constituents and DNA gyrase [76], identify potential targets but do not demonstrate biochemical inhibition.

**Figure 2:**
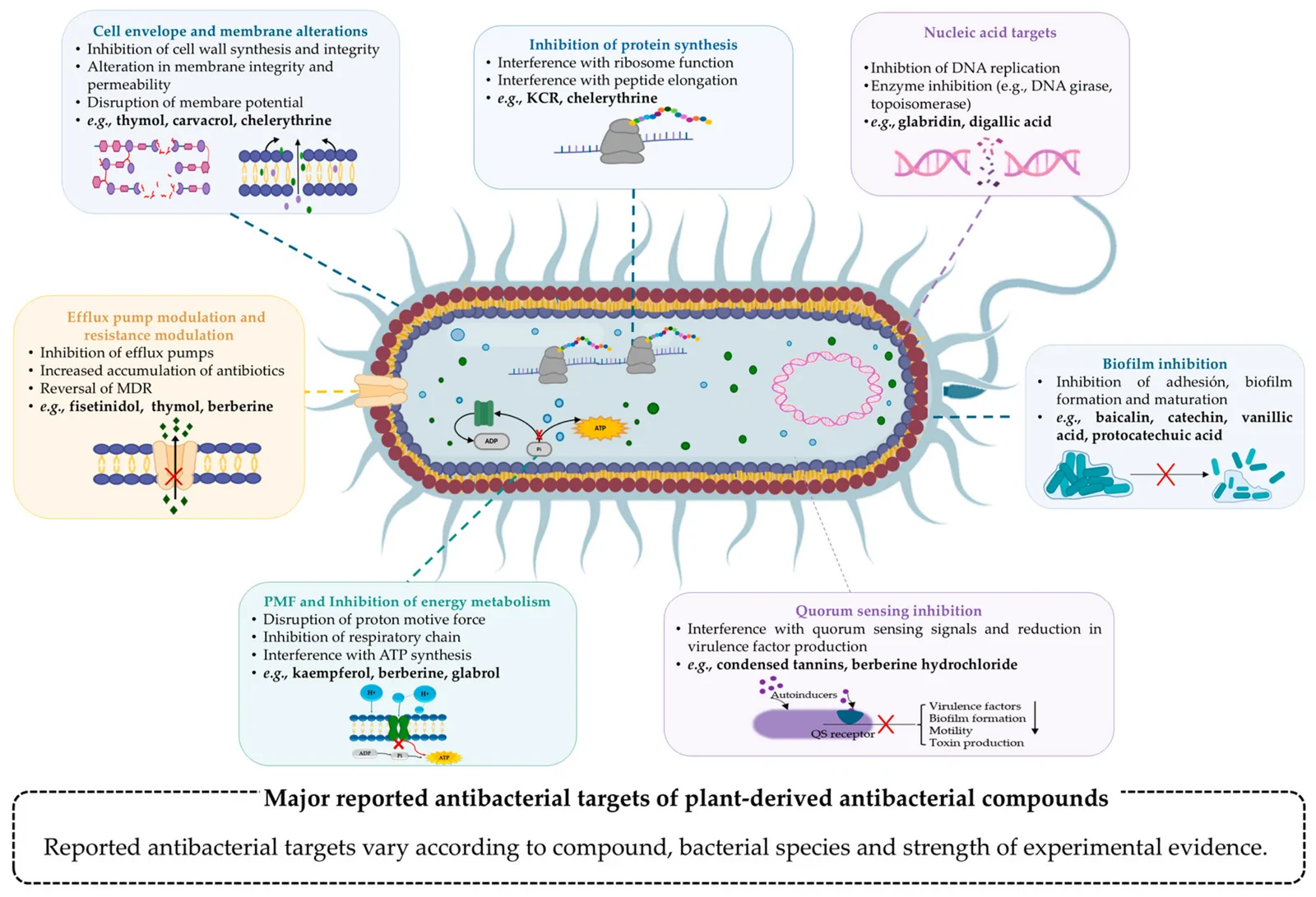
Major reported antibacterial targets of plant secondary metabolites. The mechanisms shown represent bacterial processes affected by representative compounds and should not be interpreted as universally demonstrated or simultaneously occurring mechanisms. The strength of experimental evidence varies among compounds and studies. Colored dashed lines connect each mechanism to its site of action in the bacterial cell. Red crosses indicate inhibition or blockade. The colored boxes distinguish the different mechanistic categories.

Mechanistic attribution is particularly challenging for essential oils, extracts, and other multicomponent preparations, because their antibacterial effects may result from additive, synergistic, or antagonistic interactions among constituents. Therefore, mechanisms demonstrated for complex preparation should not be automatically assigned to its major compound. Overall, the most robust mechanistic conclusions arise when complementary approaches converge on the same cellular target, and computational or omics-based observations are supported by functional experiments. Figure 2 summarizes the major reported bacterial targets of plant secondary metabolites, whereas Table 1 integrates representative antibacterial activity data with the principal mechanistic findings reported for selected compounds.

## 6. Pharmacokinetics

Pharmacokinetic characterization is essential for evaluating whether plant-derived antibacterial compounds can reach and maintain concentrations compatible with their in vitro activity. Absorption, distribution, metabolism, and elimination determine systemic and tissue exposure, while chemical structure, formulation, plasma protein binding, intestinal transport, and interactions with metabolic enzymes can substantially modify the pharmacokinetic behavior of natural products [78,79,80]. These factors represent important barriers to the clinical translation of phytochemicals, particularly when low bioavailability or structural instability limits exposure to the bioactive molecular species [81].

For antibacterial development, pharmacokinetic parameters should therefore not be interpreted independently from pharmacodynamic activity. Ideally, exposure to the active parent compound and relevant metabolites should be related to antibacterial potency, tissue penetration, and safety. This distinction is particularly important for plant-derived products because pharmacokinetic studies may evaluate isolated compounds, standardized extracts, or multicomponent preparations with substantially different exposure profiles [79,80,82,83].

### 6.1. Absorption and Oral Bioavailability

Oral absorption of plant-derived compounds varies substantially according to chemical structure, solubility, formulation, intestinal transport, metabolism, and the administered matrix [79,80]. Poor or variable bioavailability remains a recurrent challenge for phytochemicals and has also been recognized for antimicrobial saponins [81,84]. Importantly, rapid absorption does not necessarily imply high systemic exposure or oral bioavailability. Therefore, parameters such as time to maximum plasma concentration (T_max_) should be interpreted together with C_max_, AUC, and absolute or relative bioavailability when these parameters are available.

Berberine provides a relevant example of the discrepancy between antibacterial activity and systemic exposure. Although berberine has demonstrated antibacterial activity against clinically relevant bacterial isolates [46,47], pharmacokinetic analysis in rats showed an absolute oral bioavailability of only 0.37 ± 0.11% following oral administration, with extensive metabolism contributing to the low circulating exposure to the unchanged parent compound [85]. Thus, antibacterial activity measured for berberine in vitro should not be assumed to correspond directly to concentrations of unchanged berberine achieved systemically.

Quercetin shows a similarly complex relationship between administered compound and circulating molecular species. Following oral administration of quercetin in rats, quercetin-3-O-β-glucuronide (Q3G) represented a substantially greater component of systemic exposure than unchanged quercetin, and the plasma profiles exhibited multiple concentration maxima consistent with extensive absorption and metabolic interconversion [86]. This distinction is relevant because quercetin has demonstrated antibacterial and membrane-associated effects [54,70], whereas the antibacterial activity of its circulating conjugates cannot automatically be assumed to be equivalent to that of the parent aglycone.

Baicalin provides another antibacterial-relevant example. Pharmacokinetic studies have reported delayed absorption and double-peak plasma profiles consistent with intestinal reabsorption and enterohepatic recirculation [15,16]. Baicalin has demonstrated antibacterial and antivirulence activity against MRSA, including reduced bacterial burden in an infected-mouse model [14]; nevertheless, these pharmacokinetic observations do not establish that systemically available concentrations reproduce the antibacterial concentrations used in vitro.

Additional pharmacokinetic studies of phenolic compounds, alkaloids, and multicomponent botanical preparations further demonstrate substantial variation in intestinal absorption [87,88,89,90]. However, such data should not be extrapolated directly to purify antibacterial compounds when the administered material differs in chemical composition or formulation.

### 6.2. Distribution and Tissue Exposure

Following absorption, plant-derived compounds may undergo broad systemic distribution or preferentially accumulate in specific organs. Tissue distribution is influenced by physicochemical properties, tissue perfusion, membrane permeability, and plasma protein binding [79]. However, tissue detection alone does not establish pharmacologically relevant exposure. For antibacterial applications, the concentration achieved at the site of infection, the unbound fraction, and the duration of exposure are more informative than only presence of a compound in a tissue [91].

The pharmacokinetic behavior of quercetin illustrates the importance of identifying the molecular species reaching tissues. Following administration, Q3G was detected in the kidneys, liver, heart, and brain, whereas systemic exposure after oral quercetin administration was dominated by the conjugated metabolite rather than unchanged quercetin [86]. Consequently, tissue exposure to quercetin-related species cannot be interpreted as equivalent to exposure to the aglycone used in antibacterial assays.

Distribution may also vary substantially among plant-derived compounds and preparations. Wogonin showed broad tissue distribution, with preferential accumulation in the liver and kidneys [92]. Similarly, constituents from *S. diffusa* were detected in the heart, liver, spleen, lungs, and kidneys following oral administration [17]. Although these findings demonstrate that the measured compounds or constituents reach multiple tissues, they do not establish whether the concentrations achieved are sufficient to reproduce the antibacterial effects observed in vitro.

Beyond tissue localization, the fraction of compound that remains pharmacologically available is particularly relevant. Palmatine provides a complementary example in which pharmacokinetics, tissue distribution, and plasma protein binding have been characterized for the isolated compound. Following intragastric and intravenous administration in rats, palmatine was detected in multiple tissues, with relatively high concentrations in the liver and kidney, while plasma protein binding was also characterized [93]. Because palmatine has demonstrated antibacterial activity [50], these data are relevant to its antibacterial development; nevertheless, total tissue or plasma concentrations do not necessarily represent the unbound concentration available to interact with bacterial targets. Consequently, total measured concentrations may overestimate pharmacologically relevant exposure, particularly when pharmacokinetic parameters are compared with MIC values [91].

Interpretation becomes further complicated when pharmacokinetic studies evaluate multicomponent preparations rather than isolated compounds. Chelerythrine, which has demonstrated antibacterial activity and membrane-associated effects [48,49], has undergone pharmacokinetic and metabolic characterization. Huang et al. detected chelerythrine in several tissues following repeated oral administration of *Macleaya cordata* extract [94]. Because the tissue-distribution experiment involved a multicomponent extract, these findings should not be interpreted as equivalent to the distribution of orally administered isolated chelerythrine. Similarly, alkaloids from *Corydalis yanhusuo* (Y.H.Chou & Chun C.Hsu) W.T.Wang ex Z.Y.Su & C.Y.Wu exhibited marked liver distribution following transdermal administration [95], but these findings apply to the evaluated alkaloid preparation and route of administration rather than to alkaloids as a chemical class.

Collectively, these studies show that antibacterial interpretation of tissue distribution requires consideration of the molecular species measured, tissue concentration, unbound fraction, administered preparation, and route of administration. Broad tissue distribution may favor access to infected compartments, but translational relevance ultimately requires quantitative comparison between pharmacologically available exposure at the infection site and antibacterial potency. In the absence of such data, tissue accumulation should be interpreted as evidence of distribution rather than antibacterial efficacy.

### 6.3. Metabolism

Metabolic transformation can substantially modify the systemic exposure of plant-derived compounds and should therefore be considered when interpreting their antibacterial potential. Plant-derived natural products may undergo extensive phase I and phase II metabolism, while intestinal and microbial metabolism can further modify the molecular species reaching systemic circulation [79,80]. Consequently, antibacterial activity measured for a parent compound in vitro should not automatically be extrapolated to the molecular species predominantly present in vivo.

Berberine and quercetin illustrate how metabolism can markedly alter the relationship between the administered compound and systemic exposure. Feng et al. quantified berberine together with nine phase I and phase II metabolites after intravenous and oral administration in rats and demonstrated extensive metabolic conversion, with conjugated metabolites contributing substantially to systemic exposure [85]. Similarly, following oral administration of quercetin, (Q3G) represented a substantially greater component of systemic exposure than unchanged quercetin [86]. Because the antibacterial activities discussed for berberine and quercetin have primarily been characterized for the parent compounds [46,47,54,70], equivalent activity of their circulating metabolites should not be assumed without direct experimental evidence.

Chelerythrine provides a complementary example involving a different metabolic pathway. Twelve metabolites were characterized in rat liver S9 preparations and in vivo, with reduction of the iminium bond representing an important metabolic transformation [94]. Chelerythrine has demonstrated antibacterial activity in separate microbiological studies [48,49]; however, the antibacterial activities of its individual metabolites have not been established to be equivalent to that of the parent compound. This example further illustrates that identification of metabolic products does not by itself establish their pharmacodynamic relevance.

A related but distinct metabolic consideration is the ability of some plant-derived antibacterial compounds to modify drug-metabolizing pathways. Berberine has been shown to suppress CYP3A4 transcription through downregulation of the pregnane X receptor (PXR) and to accelerate CYP3A4 protein degradation through polyubiquitination [96]. Such effects may alter the disposition of co-administered drugs and should therefore be considered when berberine or other plant-derived antibacterial compounds are evaluated in combination with conventional therapies.

Metabolic interconversion between conjugated and aglycone forms represents another important consideration, particularly for flavonoids. Pharmacokinetic studies of *Scutellariae Radix* have evaluated baicalin, wogonoside, and oroxylin A 7-*O*-glucuronide together with their corresponding aglycones [15], while additional experimental work demonstrated differences in the predominant forms of wogonin and wogonoside between plasma and intestinal or hepatic compartments [78]. Human pharmacokinetic studies of baicalein have likewise demonstrated extensive formation of glucuronide and sulfate conjugates following oral administration [97]. These observations indicate that systemic exposure may involve several structurally related molecular species rather than a single unchanged compound and that their relative contribution may differ among biological compartments.

Collectively, these examples emphasize that parent compounds and their metabolites may differ in antibacterial potency, distribution, persistence, and other pharmacokinetic properties. Therefore, meaningful exposure–response assessment requires identification of the molecular species present in plasma and relevant tissues and, where possible, independent determination of their antibacterial activity. Modern mass-spectrometry-based approaches increasingly permit simultaneous characterization of parent compounds and metabolites, but integration of these data with pharmacodynamic outcomes remains limited in natural-product research [80].

Overall, metabolic characterization should establish not only how extensively a plant-derived antibacterial compound is transformed but also which parent and metabolite species are present at pharmacologically relevant sites. Antibacterial relevance therefore requires linking exposure to individual molecular species with their corresponding antibacterial activities rather than relying solely on the pharmacokinetic profile of the administered parent compound.

### 6.4. Excretion

Elimination characteristics vary considerably among plant-derived compounds and influence the duration of systemic exposure. Excretion may occur through renal, biliary, intestinal, and other pathways, whereas chemical structure, metabolism, and formulation contribute to differences in clearance and persistence [79]. Consequently, exposure duration cannot be inferred solely from absorption characteristics.

Excretion studies also illustrate the importance of distinguishing the parent compound from its metabolites. Following oral administration of berberine in rats, Feng et al. quantified the recovery of the parent compound and several metabolites in urine, bile, and feces [85]. These findings demonstrate that elimination involves both unchanged berberine and metabolically transformed species and that recovery of the parent compound alone does not adequately describe its disposition. Alkaloids from *Corydalis bungeana* further illustrate differences in persistence, with elimination half-lives ranging from 6.85 to 9.44 h following oral administration of the extract [90]. However, these values apply to the measured alkaloids within the specific preparation and should not be generalized to other alkaloids or formulations.

Wogonin is eliminated through multiple routes; following intravenous administration in rats, approximately 21% of the administered dose was recovered unchanged in feces, urine, and bile [92]. Because this value represents recovery of unchanged wogonin, it should not be interpreted as a complete mass balance of the administered dose, particularly in the presence of metabolic interconversion with conjugated forms. Chelerythrine likewise undergoes extensive metabolism, with multiple metabolites identified in excreta following intragastric administration in rats [94]. These observations reinforce the need to distinguish elimination of the parent compound from elimination of its metabolites when interpreting systemic persistence.

Human pharmacokinetics data provides a complementary example. Following repeated oral administration of baicalein, the parent compound and its conjugated metabolites were detected in urine, and a bimodal urinary excretion profile was observed during the sampling period [97]. Such findings characterize the temporal pattern of elimination but do not, by themselves, establish the duration of antibacterial activity.

Systemic exposure may also be prolonged or re-established through secondary pharmacokinetic processes. Baicalin has shown double-peak plasma concentration profiles that have been associated with intestinal reabsorption and/or enterohepatic recirculation [16]. Such processes may extend exposure beyond the initial absorption phase. However, the presence of secondary plasma peaks or prolonged residence should not be interpreted as sustained antibacterial activity unless the molecular species present during these later phases remain at pharmacodynamically relevant concentrations.

Overall, excretion, elimination of half-life, and recirculation contribute to the duration of exposure to plant-derived antibacterial compounds, but none of these parameters independently establish antibacterial persistence. Translational interpretation requires determining which active parent compounds or metabolites remain in systemic circulation or relevant tissues, for how long, and whether their concentrations remain compatible with antibacterial activity.

### 6.5. Pharmacokinetic/Pharmacodynamic Integration and Translational Relevance

The translational relevance of plant-derived antibacterial compounds depends not only on their in vitro potency but also on whether pharmacologically active concentrations can be achieved and maintained at the site of infection. This remains a major challenge in natural-product development because limited bioavailability, metabolic transformation, formulation-dependent exposure, and structural instability may restrict the translation of promising biological activity into therapeutic efficacy [79,80,81].

Importantly, low systemic bioavailability does not necessarily imply low therapeutic efficacy, because antibacterial activity may depend on local exposure, preferential distribution to the infected tissue, or the formation of pharmacologically active metabolites [79,80]. Therefore, the relevant exposure should correspond to the active molecular species present at the site of infection rather than exclusively to the circulating concentration of the administered parent compound [78,80,91].

In antibacterial pharmacology, pharmacokinetic/pharmacodynamic (PK/PD) analysis integrates drug exposure with bacterial susceptibility. The principal exposure-based indices used for conventional antibacterial agents include the ratio of the free maximum concentration to the minimum inhibitory concentration (fC_max_/MIC), the ratio of the free 24-h area under the concentration–time curve to the MIC (fAUC_24_/MIC), and the percentage of the dosing interval during which free drug concentrations remain above the MIC (fT>MIC) [91]. These indices are associated with different temporal patterns of antibacterial activity and provide a quantitative framework for linking exposure with bacterial response.

However, PK/PD relationships established for conventional antibiotics should not be directly assigned to plant-derived compounds according to their chemical class or proposed mechanism of action. Identification of the PK/PD index that best predicts antibacterial efficacy requires experimental characterization of the concentration–effect relationship. Moreover, MIC is a static endpoint determined at a defined observation time and does not capture the temporal dynamics of bacterial growth and killing. Longitudinal pharmacodynamic experiments, time-kill analyses, and pharmacometric modeling can therefore provide information that is not captured by MIC alone [98].

Another critical consideration is that PK/PD relationships should ideally be based on pharmacologically relevant exposure rather than total measured concentrations. Plasma protein binding, tissue penetration, metabolism, and the formation of active or inactive metabolites can substantially modify the exposure experienced by bacteria. Accordingly, total plasma or tissue concentrations may not represent the freely available concentration at the infection site. Comparison of an in vitro MIC with total plasma C_max_ or AUC should therefore be regarded as preliminary unless the unbound fraction and relevant tissue exposure are known [91].

Available cross-study comparisons illustrate the magnitude of this translational gap, although they should not be interpreted as validated PK/PD relationships. For berberine, oral administration of 48.2–240 mg/kg in rats produced parent-compound C_max_ values of approximately 4.11–32.6 ng/mL [85], whereas antibacterial studies reported MIC values of approximately 64–256 µg/mL against clinical MRSA isolates [46]. Thus, even the highest reported total plasma C_max_ was approximately 2000–8000-fold lower than these MIC values. Similarly, repeated oral administration of 600 mg baicalein in healthy subjects produced a total steady-state C_max_ of approximately 1.32 µg/mL [97], whereas baicalein MIC values of 0.5–1.5 mmol/L, equivalent to approximately 135–405 µg/mL, were reported against the bacterial panel evaluated in a separate study [54]. These MIC values were therefore approximately 100–300-fold higher than the observed total plasma C_max_. Importantly, these comparisons involve different experimental studies and total rather than unbound plasma concentrations, and neither study quantified active concentrations at an infection site. They should therefore be interpreted as preliminary exposure–potency comparisons rather than evidence of therapeutic failure or validated PK/PD relationships.

PK/PD integration is particularly challenging for plant-derived antibacterials because pharmacokinetic and antibacterial studies frequently evaluate different preparations or molecular species. Pharmacokinetic exposure to an isolated constituent should not be directly compared with the MIC of a complex extract or essential oil, and exposure to a parent compound cannot automatically be assumed to represent exposure to its metabolites. Furthermore, metabolic transformation may produce molecular species with biological and pharmacokinetic properties different from those of the administered compound [79,80]. Meaningful PK/PD analysis therefore requires pharmacokinetic exposure and antibacterial potency to be determined for the same chemically defined compound or preparation. The importance of identifying the molecular species present after administration is consistent with the recent natural-product bioavailability and metabolism literature.

In complex plant preparations, interactions among constituents may influence both pharmacokinetic exposure and pharmacodynamic activity. Co-occurring compounds may modify absorption, metabolism, distribution, or elimination of other constituents, thereby increasing or decreasing their effective exposure. At the pharmacodynamic level, constituent interactions may be synergistic, additive, or antagonistic, so the activity of the complete preparation cannot be predicted solely from the concentration or activity of its major components. However, quantitative evidence linking such interactions to antibacterial PK/PD remains limited, and these effects should therefore be demonstrated experimentally rather than assumed [24,79,80].

Although preliminary cross-study exposure–potency comparisons can be made for selected compounds, the studies summarized in this review predominantly provide conventional pharmacokinetic parameters rather than prospectively matched antibacterial PK/PD data. Paired quantitative relationships between unbound exposure at the infection site and antibacterial effect therefore remain scarce. Consequently, validated PK/PD targets cannot currently be proposed for most of the plant-derived antibacterial compounds discussed here. This represents a major translational gap. Recent evaluations of phytochemical and plant-derived antibacterial development similarly identify poor bioavailability, insufficient preclinical characterization, and limited progression from promising antibacterial hits to advanced drug-development stages as persistent barriers [80,81,99].

Thus, this section discusses berberine, quercetin, palmatine, chelerythrine, and baicalein because both antibacterial activity and pharmacokinetic data are available for these chemically defined compounds. Nevertheless, quantitative PK/PD relationships remain insufficiently established, and pharmacokinetic exposure should not be interpreted as evidence of antibacterial efficacy in the absence of corresponding concentration–effect data.

**Figure 3:**
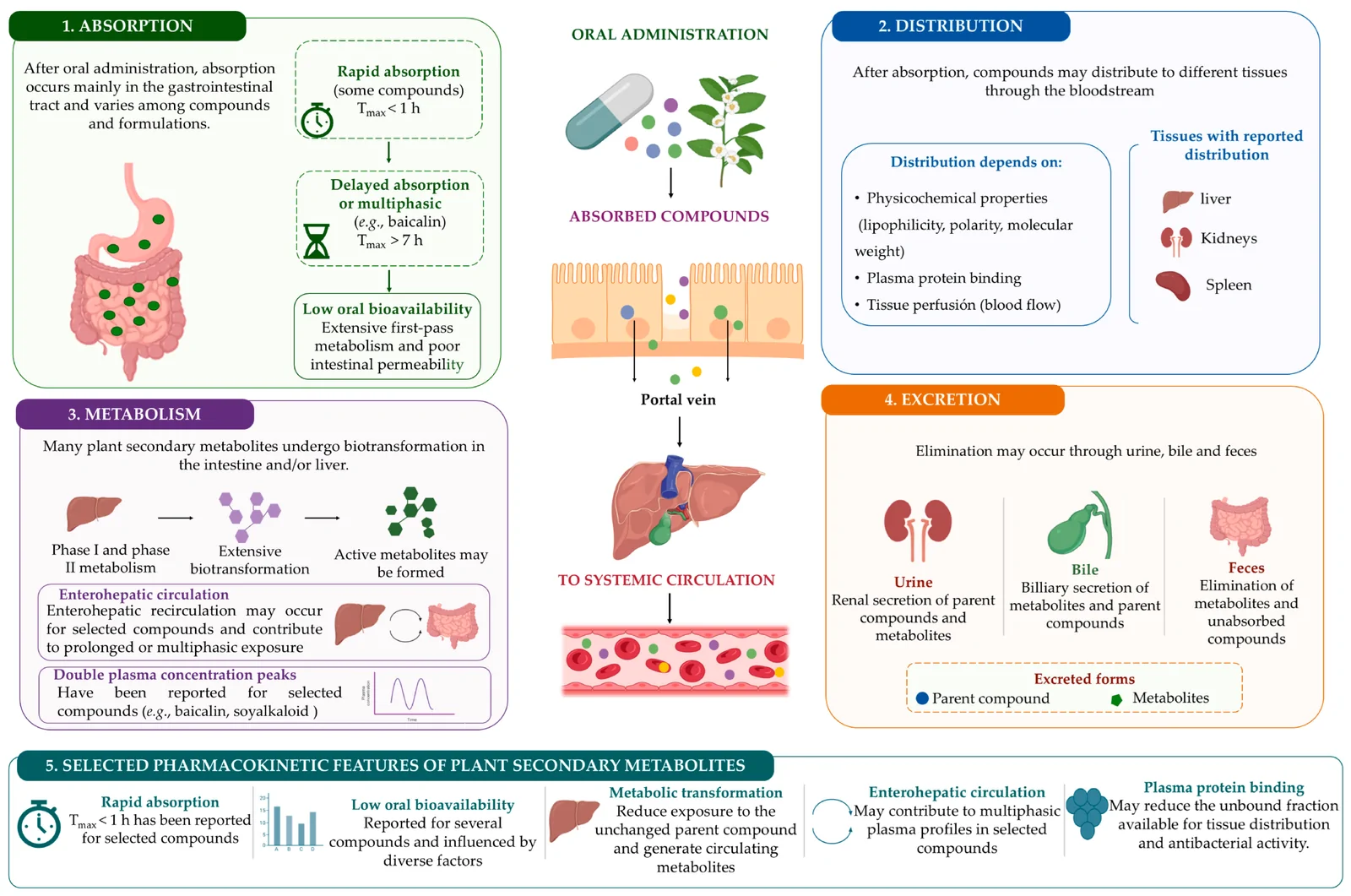
Schematic overview of pharmacokinetic processes and selected pharmacokinetic features reported for antibacterial plant secondary metabolites following oral administration. Absorption, distribution, metabolism, and elimination vary according to the chemical characteristics of the compound, formulation, route, and experimental model. The features illustrated represent findings reported for selected compounds and should not be interpreted as universal pharmacokinetic properties of all plant secondary metabolites. Importantly, systemic exposure does not necessarily indicate that antibacterial concentrations are achieved at the site of infection.

Future studies should therefore integrate quantitative antibacterial assays with plasma and tissue pharmacokinetics, determine the contribution of parent compounds and pharmacologically active metabolites, and characterize concentration- and time-dependent bacterial killing. Dose-fractionation studies, dynamic in vitro infection models, and appropriate in vivo models could subsequently be used to identify the PK/PD index and magnitude associated with bacterial stasis or killing. When sufficient data becomes available, pharmacometric modeling and probability-of-target-attainment analyses could determine whether achievable exposures are compatible with antibacterial efficacy while remaining within acceptable safety limits [91,98]. Such integration would provide a more rigorous basis for translating promising in vitro plant-derived antibacterials into viable therapeutic candidates. Representative pharmacokinetic findings and the main limitations affecting the translation of plant-derived antibacterial compounds are summarized in Table 2. Figure 3 provides a schematic overview of the principal pharmacokinetic processes and selected features reported for these compounds.

## 7. Safety, Selectivity, and Translational Considerations

### 7.1. In Vitro Cytotoxicity and Antibacterial Selectivity

Available studies indicate that antibacterial activity and host–cell toxicity can occur within overlapping concentration ranges for some plant-derived compounds. Therefore, cytotoxicity should preferably be evaluated in parallel with antibacterial potency using relevant mammalian cell models. When both parameters are available, the selectivity index (SI), commonly expressed as the ratio between the 50% cytotoxic concentration (CC_50_) and the MIC, provides a useful preliminary estimate of the therapeutic window, although its interpretation remains dependent on the bacterial strain, cell model, exposure time, and assay conditions. This comparison is relevant for membrane-active compounds, because antibacterial membrane disruption may occur at concentrations close to those affecting mammalian-cell viability [100,101,102,103].

Carvacrol illustrates the importance of this comparison. In a study, carvacrol showed MIC values of 100 µg/mL against the bacterial strains evaluated and did not significantly reduce cell viability at concentrations ≤ 150 µg/mL in murine peritoneal macrophages and L929 fibroblasts. The corresponding CC_50_ values were 208.30 ± 63.73 and 265.51 ± 36.05 µg/mL, respectively [104]. These data indicate measurable separation between antibacterial and cytotoxic concentrations, although the margin remained relatively narrow and depended on the cell model.

A different pattern was reported when thymol and carvacrol were tested against bacterial and fungal pathogens together with human epithelial cell lines. Their antibacterial MIC/MBC values generally ranged from 125 to 250 µg/mL, whereas significant cytotoxicity was observed at lower concentrations in some cell models, particularly for carvacrol [100]. Thus, apparently similar antibacterial potency can be associated with markedly different host–cell responses depending on the biological model and exposure conditions. These findings also illustrate why safety conclusions should not be generalized from one cell type or assay.

Berberine provides another example in which antibacterial and cytotoxic concentration ranges partially overlap. Against 50 clinical MRSA isolates, berberine exhibited MIC values ranging from 32 to 256 µg/mL. In human MRC-5 fibroblasts and FHC epithelial cells, concentrations of 16–64 µg/mL did not significantly affect cell proliferation, whereas cytotoxic effects became evident at concentrations of 128 µg/mL and above [101]. Consequently, some MRSA isolates were inhibited within concentrations that showed limited effects on the tested human cells, whereas higher-MIC isolates required concentrations approaching or exceeding the cytotoxic range. This illustrates a strain-dependent relationship between antibacterial potency and host–cell selectivity.

Formal selectivity analysis has also been applied to plant-derived phenolics. Isobavachalcone showed an MIC of 5 µM against *S. aureus* ATCC 29213 and a CC_50_ of 94 µM in RAW 264.7 macrophages, corresponding to an SI of 19. A structurally related derivative exhibited an SI of 23, whereas another derivative with broader antibacterial activity showed an SI of only 5 [102]. These findings demonstrate that structural modifications capable of altering antibacterial potency can also substantially modify mammalian-cell toxicity, reinforcing the need to integrate structure–activity and structure–toxicity relationships during optimization.

The available evidence indicates that host–cell selectivity varies substantially among plant-derived antibacterial materials, target microorganisms, and experimental models [103,105]. Cytotoxicity should therefore be interpreted relative to the concentration required for antibacterial activity using the same chemically defined material and comparable experimental conditions [103]. Antibacterial potency without corresponding host–cell toxicity data provides incomplete evidence of therapeutic potential, whereas low cytotoxicity observed only at concentrations below the antibacterial range does not establish a meaningful safety margin [103,105,106].

### 7.2. In Vivo Safety and Tolerability

In vitro cytotoxicity provides an initial estimate of host–cell selectivity but cannot fully characterize systemic safety. In vivo toxicological assessment can reveal dose- and exposure-dependent effects that are not captured by cell-based assays, including alterations in clinical behavior, body or organ weight, hematological and biochemical parameters, and tissue histopathology [107,108,109]. The absence of mortality alone should not be interpreted as evidence of safety, because non-lethal doses may still produce organ-specific or subclinical toxicity [107,109].

Baicalin provides an example of dose-dependent systemic safety. In an acute oral toxicity study, a single dose of 4000 mg/kg produced no mortality in mice, corresponding to an LD_50_ above 4000 mg/kg [107]. However, in a 28-day repeated-dose study, daily administration of 4000 mg/kg produced reversible hepatic and renal alterations, whereas the authors established a no-observed-adverse-effect level (NOAEL) of 2000 mg/kg/day [107]. These findings illustrate that absence of acute mortality does not exclude toxicity following repeated exposure.

Formulation may also influence the toxicological profile and should therefore be specified when interpreting safety data. Berberine-loaded liposomes were evaluated in rats using acute and 28-day repeated-dose oral toxicity protocols. No acute toxicity was observed at doses equivalent to 2000 and 5000 mg/kg of berberine, and repeated administration at 150 or 450 mg/kg/day for 28 days produced no significant changes in general condition, body or organ weights, hematological or biochemical parameters, or histological findings [108]. However, these results apply specifically to liposomal formulation and should not be extrapolated directly to unformulated berberine.

Essential oils provide a contrasting example in which non-lethal repeated exposure can still produce organ-specific toxicity. In rats, a single oral dose of 2000 mg/kg of *Thymus vulgaris* L. essential oil caused marked clinical signs of toxicity and mortality in two of three animals, whereas 300 mg/kg produced only transient manifestations [109]. During repeated administration for 28 days, all animals survived at 100, 250, and 500 mg/kg/day, and hematological and biochemical parameters remained largely unchanged. Nevertheless, 500 mg/kg/day produced severe pulmonary histopathological alterations and reduced body weight in male rats, leading the authors to propose a NOAEL above 250 mg/kg/day rather than at the highest tested dose [109].

The above-mentioned studies show that safety conclusions for plant-derived compounds should specify the tested material, formulation, route of administration, dose, and duration of exposure [107,108,109]. Moreover, toxicological interpretation should incorporate organ-specific, biochemical, hematological, and histopathological endpoints rather than relying exclusively on survival or gross clinical observations. Recent evaluations of medicinal and aromatic plants similarly emphasize that hepatotoxicity, nephrotoxicity, neurotoxicity, genotoxicity, and other adverse effects can occur with plant-derived materials and require systematic assessment during therapeutic development [110].

### 7.3. Safety Limitations and Translational Implications

The natural origin of a plant-derived compound should not be interpreted as evidence of biological safety. Medicinal and aromatic plant products can produce adverse effects involving hepatic, renal, gastrointestinal, neurological, and other organ systems, and genotoxic or reproductive effects have also been reported for some plant-derived materials [110]. Safety conclusions must therefore be established experimentally for the specific compound, extract, essential oil, or formulation under investigation rather than extrapolated from traditional use or from other preparations derived from the same plant species [106,110].

A further limitation is that in vitro cytotoxicity and selectivity indices provide only an initial estimate of therapeutic safety. Recent analyses of essential oils against both Gram-positive and Gram-negative ESKAPE/ESKAPEE pathogens identified materials with wide selectivity margins but also preparations with low selectivity indices, indicating that antibacterial concentrations may approach concentrations that are cytotoxic to mammalian cells [105,106]. Moreover, the calculated selectivity can vary according to the microorganism and mammalian cell model used. Consequently, favorable in vitro selectivity should be considered a criterion for prioritizing candidates for further investigation rather than definitive evidence of in vivo safety [105,106].

Translation is additionally complicated by the interaction between safety, pharmacokinetics, formulation, and chemical standardization. Poor bioavailability, metabolic transformation, structural instability, and inadequate preclinical models can alter the exposure to phytochemicals and limit the extrapolation of in vitro findings to therapeutic conditions [81]. In plant-derived antibacterial development specifically, recent analyses have identified bioavailability, pharmacokinetic characterization, toxicity and selectivity, standardization of plant preparations, formulation, and limited in vivo validation as major barriers to progression toward clinical application [99,111]. Therefore, toxicological results obtained for one formulation or preparation should not automatically be extrapolated to another with different composition or pharmacokinetic behavior.

An additional challenge concerns the distinction between isolated compounds and chemically complex preparations. Unlike purified molecules, extracts and essential oils may show substantial compositional variability depending on plant material, extraction procedures, and preparation conditions, which can affect antibacterial activity, pharmacokinetics, and experimental reproducibility. Their biological effects may also reflect interactions among multiple constituents rather than the activity of the quantitatively predominant component. Consequently, attribution of antibacterial activity to a major constituent requires direct experimental comparison with the isolated compound and, ideally, with defined combinations of relevant constituents [23,25,26,27,79,111].

Assessment of the therapeutic potential of plant-derived antibacterials should integrate antibacterial potency, host–cell selectivity, pharmacokinetic exposure, repeated-dose toxicity, and efficacy in appropriate infection models. A low MIC or MBC alone is insufficient if the effective concentration cannot be achieved at the infection site without approaching toxic exposure. Conversely, favorable cytotoxicity data have limited translational significance when they are obtained at concentrations below those required for antibacterial activity. Recent translational frameworks for plant-derived antibacterial agents similarly emphasize a stepwise progression from in vitro activity and mechanistic characterization to in vivo validation, PK/PD and toxicological assessment, formulation optimization, and ultimately clinical evaluation [111]. Representative evidence on host–cell selectivity and in vivo safety of plant-derived antibacterial compounds and preparations is summarized in Table 3.

## 8. Recent Mechanistic Advances in Plant-Derived Antibacterials (2024–2026)

Recent studies have increasingly moved beyond descriptive observations of bacterial damage toward functional characterization of specific antibacterial processes, resistance-associated mechanisms, and resistance-development phenotypes. In 2024, garlic essential-oil nanoemulsion against *L. monocytogenes* produced membrane depolarization, loss of membrane integrity, and leakage of nucleic acids, proteins, and ATP, providing functional evidence of membrane-associated antibacterial activity [65]. More target-specific evidence emerged in 2025. Carvacrol showed MIC values of 4–16 µg/mL against a panel of drug-resistant bacteria and affected FtsZ polymerization, supporting disruption of bacterial cell division as an additional antibacterial mechanism; molecular modeling further suggested interaction with the FtsZ T7-loop, although this latter evidence remains predictive [112]. In parallel, thymol and carvacrol were shown to affect efflux-associated processes and resistance development in *A. baumannii*. During 30-day serial-passage experiments, neither compound produced substantial increases in MIC when used alone, whereas combinations with aminoglycosides attenuated the increase in antibiotic resistance observed during prolonged exposure [67].

Mechanistic evidence expanded further in 2026. *Origanum vulgare* L. essential oil and its major constituent’s carvacrol and thymol showed antibacterial and antibiofilm activity against clinical carbapenem-resistant *A. baumannii*, with MIC and MBC values of 128–512 µg/mL. These effects were accompanied by membrane damage, reduced ATP levels, increased ROS, and transcriptomic alterations involving fatty-acid metabolism, metal-ion homeostasis, and xenobiotic degradation; the transcriptomic findings should nevertheless be regarded as supportive rather than as direct evidence of specific target inhibition [113]. Berberine hydrochloride was also evaluated against multidrug-resistant *A. baumannii*, where its combination with antibiotics was associated with altered expression of several efflux-related genes, including *adeR*, *adeS*, *adeJ*, *adeM*, *adeG*, and *adeB*, providing recent evidence of resistance modulation linked to efflux regulation [114].

Particularly strong functional evidence was reported for quercetin as a tigecycline adjuvant against multidrug-resistant *A. baumannii*. Quercetin reduced tigecycline MIC values by 4- to 32-fold, increased intracellular tigecycline concentrations measured by HPLC, and was associated with increased membrane permeability and inhibition of RND-mediated efflux. Importantly, the combination also exhibited antibacterial efficacy in a murine pulmonary infection model [115]. Finally, a carvacrol–thymol combination showed synergistic bactericidal activity against *S. aureus* and *E. coli* without a detectable progressive increase in MIC during 96 h of subinhibitory exposure. Because this study used a short-term model and reference strains, these findings support absence of early resistance induction under the tested conditions rather than definitive evidence of long-term resistance prevention [116].

Collectively, these studies illustrate a recent progression from general descriptions of membrane damage toward experimentally supported bacterial targets, direct assessment of intracellular antibiotic accumulation, efflux-associated resistance mechanisms, and resistance-development experiments. However, the strength of evidence remains heterogeneous: direct functional measurements provide stronger mechanistic support than transcriptomic associations or computational predictions, and most of these findings have not yet been linked to quantitative PK/PD relationships or validated across multiple in vivo infection models.

## 9. Conclusions and Future Perspectives

Plant-derived antibacterial compounds encompass chemically and experimentally heterogeneous materials, ranging from purified molecules to essential oils, fractions, and extracts. The evidence reviewed here indicates that these materials can affect multiple bacterial processes, including cell-envelope integrity, membrane potential and permeability, energy metabolism, protein synthesis, multidrug efflux, quorum sensing, biofilm formation, and virulence-associated pathways [22,31,34,35,44,47,62,67]. However, the strength of mechanistic evidence varies considerably. Functional measurements of membrane dysfunction, enzyme activity, translation, efflux, or signaling provide stronger mechanistic support than morphological alterations, transcriptomic or metabolomic associations, and molecular docking considered in isolation.

The occurrence of multiple antibacterial effects should nevertheless not be interpreted as evidence that plant-derived compounds are intrinsically less prone to resistance development. Experimental evidence supporting resistance prevention remains compound- and condition-specific. For example, thymol and carvacrol combinations have been evaluated under prolonged antibacterial exposure and resistance-development conditions [67], but such findings cannot be generalized to all terpenoids, phenolics, alkaloids, or complex preparations. Multitarget activity should be considered a mechanistic characteristic with potential relevance to resistance evolution rather than proof of a universally lower resistance risk.

Pharmacokinetic evidence further demonstrates that in vitro antibacterial activity alone does not necessarily translate into therapeutically relevant exposure. Absorption, bioavailability, distribution, protein binding, metabolism, and elimination vary substantially among plant-derived compounds and formulations [79,80]. Rapid absorption, broad tissue distribution, or prolonged elimination alone does not establish antibacterial efficacy because the relevant determinant is whether active, preferably unbound, molecular species reach the infection site at concentrations compatible with antibacterial activity. Consequently, integrated PK/PD relationships rather than isolated pharmacokinetic parameters are required to evaluate translational potential [91,98].

Safety represents an equally important translational constraint. Antibacterial potency should be interpreted together with host–cell cytotoxicity and, where possible, quantitative selectivity indices, because antibacterial and cytotoxic concentrations can overlap for some plant-derived compounds [105,106]. In vivo evaluation is also necessary because absence of acute mortality does not exclude dose- or organ-dependent toxicity during repeated exposure, as demonstrated for baicalin and *T. vulgaris* essential oil [107,109]. Moreover, safety data remains material- and formulation-specific and should not be extrapolated indiscriminately between purified compounds, essential oils, extracts, or delivery systems [110,111].

Direct in vivo antibacterial efficacy remains substantially less documented than in vitro antibacterial activity. Among the compounds discussed in this review, baicalin provided the clearest integrated example: it inhibited MRSA-associated biofilm and virulence-related phenotypes in vitro and improved survival while reducing bacterial burden in an infected-mouse model [14]. Pharmacokinetic studies have additionally characterized its absorption and double-peak plasma profiles [15,16], while toxicological evaluation identified an oral LD50 > 4000 mg/kg and a NOAEL of 2000 mg/kg/day in mice [107]. Nevertheless, matched infection-site exposure and antibacterial PK/PD data remain unavailable. Berberine has substantial in vitro antibacterial and mechanistic evidence, including effects on the cell envelope, proton motive force, biofilm formation, and antibiotic resistance-associated processes [45,46,47,71], together with pharmacokinetic evidence of very low oral bioavailability [85].

However, the evidence reviewed here did not identify a corresponding animal infection model demonstrating direct antibacterial efficacy, and available safety data included in vitro cytotoxicity as well as formulation-specific in vivo tolerability data that should not be extrapolated directly to unformulated berberine [101,108]. Thymol and carvacrol likewise show reproducible in vitro antibacterial activity and functional evidence of membrane disruption and resistance modulation [52,67], but compound-specific antibacterial PK/PD relationships and direct in vivo efficacy were not identified in the studies reviewed; available toxicity findings are predominantly in vitro and should not be conflated with safety data from whole essential oils [100,104,109].

Chelerythrine provides comparatively strong in vitro mechanistic evidence involving membrane dysfunction, cellular energetics, protein synthesis, and biofilm inhibition [48,49], whereas its pharmacokinetic and metabolic characterization remain separate from antibacterial efficacy studies [94], and direct in vivo antibacterial efficacy was not identified. Collectively, these comparisons show that the major translational gap is not the absence of in vitro antibacterial activity but the scarcity of studies integrating antibacterial potency, mechanism, pharmacokinetic exposure, toxicity, and efficacy in the same in vivo infection framework.

Future development should therefore follow an integrated and stepwise strategy. Initial antibacterial screening should establish reproducible MIC and MBC values for chemically defined materials and should be complemented by time-kill experiments and mechanistic assays capable of distinguishing direct cellular targets from secondary stress responses. Candidates showing sufficient potency should subsequently undergo host–cell cytotoxicity and selectivity assessment, resistance development assays, quantitative pharmacokinetic characterization of parent compounds and relevant metabolites, and determination of exposure–response relationships in appropriate PK/PD and infection models [81,91,98,111]. For complex extracts and essential oils, chemical standardization and explicit identification of the tested preparation are additionally required to ensure that antibacterial, toxicological, and pharmacokinetic findings refer to comparable materials [79,111].

Overall, current evidence supports plant-derived compounds primarily as a chemically diverse source of antibacterial lead rather than as established therapeutic candidates. For most compounds, translation remains limited by incomplete pharmacokinetic and PK/PD characterization, safety assessment, standardization and reproducibility, in vivo efficacy data, and clinical validation [99,111,117].

The main limitation is no longer simply the demonstration of in vitro antibacterial activity but the integration of chemical identity, mechanistic confidence, bacterial susceptibility, achievable exposure, PK/PD relationships, host–cell selectivity, systemic safety, and efficacy in relevant infection models. Addressing these components within the same development framework will be essential for identifying which plant-derived antibacterials have realistic potential to progress toward clinical application [81,99,111].

## Figures and Tables

**Table 1:** Antibacterial activity, bacterial targets, and mechanistic evidence for representative plant-derived antibacterial materials.

Chemical Class	Compound	Bacterial Species or Strain	MIC	Antibacterial Outcome/ Mechanistic Findings	Reference
Phenylpropanoid	eugenol	Carbapenem- resistant *K. pneumoniae*	NR	>85% killing at 0.5 mg/mL Complete eradication at 1.0 mg/mL after 24 h ROS-associated membrane damage and leakage of intracellular components	[68]
Flavonoid	Glabrol	*S. aureus* ATCC 29213; MSSA; MRSA	2 µg/mL	Complete killing at 4–16 µg/mL within 1 h Disruption of PMF and membrane permeability interaction with peptidoglycan and membrane phospholipids	[34]
Flavonoid glycoside	KCR	*S. aureus* ATCC 25923	16 µg/mL	Mechanistic experiments performed at 5× MIC Inhibition of bacterial protein synthesis	[35]
Isoflavonoid	Glabridin	*S. aureus*, *B. subtilis*, *E. coli*	8–16 µg/mL	DNA gyrase inhibition	[74]
Phenolic acid derivative	Digallic acid	*S. aureus*, MRSA; *E. coli* ATCC 25922	200 µM-NR	ATP inhibition of bacterial DNA gyrase and topoisomerase IV	[75]
Benzophenanthridine alkaloid	Chelerythrine	Carbapenem- resistant *S. marcescens*	125 µg/mL	Sub-MIC concentrations inhibited biofilm formation Disruption of membrane integrity and membrane-associated bioenergetic homeostasis	[48]
Protoberberine alkaloid	Berberine	15 clinical MRSA isolates	64–256 µg/mL	Time-kill assays Cell-wall/membrane alterations and inhibition of biofilm formation	[46]

MIC, minimum inhibitory concentration; MRSA, methicillin-resistant *Staphylococcus aureus*; MSSA, methicillin-susceptible *S. aureus*; PMF, proton motive force; ROS, reactive oxygen species; NR, not reported.

**Table 2:** Representative pharmacokinetic findings and translational considerations for plant-derived compounds with documented antibacterial activity.

Compound	Antibacterial Evidence	Model and Administration	Pharmacokinetic Findings	Limitations	Reference
Berberine	Activity against MRSA and carbapenem-resistant *P. aeruginosa*; membrane/cell-envelope effects and antibiotic-potentiating activity	Rats; oral and intravenous administration	oral bioavailability of 0.37%; parent compound and nine metabolites characterized and detected in excreta	Low oral systemic availability and extensive metabolism limit direct extrapolation of in vitro parent-compound activity to systemic exposure	[46,47,85]
Quercetin	Antibacterial activity against Gram-positive and Gram-negative bacteria; membrane-associated effects reported in *S. aureus*	Rats; oral administration of quercetin/Q3G and intravenous Q3G	Q3G represented a greater component of systemic exposure than unchanged quercetin; Q3G distributed to kidney, liver, heart, and brain	Antibacterial activity of Q3G cannot be assumed to equal that of the quercetin aglycone	[54,70,86]
Baicalin	Antibacterial and antivirulence activity against MRSA, including reduced bacterial burden in an infected-mouse model	Rats; oral botanical preparations	Delayed absorption and double-peak plasma profiles	Secondary plasma peaks may prolong or re-establish exposure, but sustained antibacterial activity requires demonstration	[14,15,16]
Palmatine	Antibacterial activity and interactions with bacterial DNA	Rats; intragastric and intravenous administration	Distribution to multiple tissues; plasma protein binding characterized	Total plasma or tissue concentrations may not reflect the unbound exposure available to interact with bacterial targets	[50,93]
Chelerythrine	Antibacterial activity against *S. aureus*, MRSA, and carbapenem-resistant *S. marcescens*; membrane and protein-synthesis effects	Rats; pharmacokinetic/metabolism studies and repeated oral administration of *M. cordata* extract	Extensive metabolism; twelve metabolites characterized; chelerythrine detected in several tissues; metabolites identified in excreta	Tissue findings obtained with a multicomponent extract should not be equated with isolated-compound exposure	[48,49,94]
Baicalein	Antibacterial activity against several bacterial species; antibiofilm activity against *S. aureus* reported	Healthy volunteers; repeated oral administration	Formation of glucuronide and sulfate conjugates; systemic exposure characterized after repeated dosing; urinary elimination of parent/conjugated species	Human PK confirms systemic exposure but does not establish attainment of antibacterial concentrations; parent and conjugate activities should be evaluated separately	[54,97]

Q3G, quercetin-3-*O-*β-glucuronide; MRSA, methicillin-resistant *Staphylococcus aureus*; PK, pharmacokinetics. Pharmacokinetic and antibacterial findings may originate from separate studies and should not be interpreted as validated PK/PD relationships unless exposure and antibacterial response were evaluated within the same experimental framework.

**Table 3:** Representative in vitro selectivity and in vivo safety evidence for plant-derived antibacterial compounds and preparations.

Compound or Material	Model	Main Safety/Selectivity Finding	Interpretation	Reference
Carvacrol	Murine macrophages and L929 fibroblasts	MIC = 100 µg/mL; CC_50_ = 208.3–265.5 µg/mL	Antibacterial activity occurred below cytotoxic concentrations but with a limited margin	[104]
Berberine	Human MRC-5 and FHC cells	MIC = 32–256 µg/mL; cytotoxic effects at ≥128 µg/mL	Antibacterial and cytotoxic ranges overlapped for higher-MIC isolates	[101]
Isobavachalcone	RAW 264.7 macrophages	MIC = 5 µM; CC_50_ = 94 µM; SI = 19	Favorable in vitro selectivity against *S. aureus* under the tested conditions	[102]
Baicalin	Mice, acute and 28-day oral exposure	LD_50_ > 4000 mg/kg; NOAEL = 2000 mg/kg/day	Repeated high-dose exposure produced reversible hepatorenal toxicity	[107]
*Thymus vulgaris* essential oil	Rats, 28-day oral exposure	Histopathological lung alterations at 500 mg/kg/day; NOAEL > 250 mg/kg/day	Survival did not exclude organ-specific toxicity	[109]

CC_50_, concentration producing a 50% reduction in host–cell viability; MIC, minimum inhibitory concentration; SI, selectivity index; LD_50_, median lethal dose; NOAEL, no-observed-adverse-effect level.

## Data Availability

No new data were created or analyzed in this study. Data sharing is not applicable to this article.
